# Self-esteem Modulates the P3 Component in Response to the Self-face Processing after Priming with Emotional Faces

**DOI:** 10.3389/fpsyg.2017.01399

**Published:** 2017-08-18

**Authors:** Lili Guan, Yufang Zhao, Yige Wang, Yujie Chen, Juan Yang

**Affiliations:** ^1^School of Psychology, Northeast Normal University Changchun, China; ^2^Faculty of Psychology, Southwest University Chongqing, China

**Keywords:** self-face processing advantage, self-esteem, emotional face prime, ERP, P3

## Abstract

The self-face processing advantage (SPA) refers to the research finding that individuals generally recognize their own face faster than another’s face; self-face also elicits an enhanced P3 amplitude compared to another’s face. It has been suggested that social evaluation threats could weaken the SPA and that self-esteem could be regarded as a threat buffer. However, little research has directly investigated the neural evidence of how self-esteem modulates the social evaluation threat to the SPA. In the current event-related potential study, 27 healthy Chinese undergraduate students were primed with emotional faces (angry, happy, or neutral) and were asked to judge whether the target face (self, friend, and stranger) was familiar or unfamiliar. Electrophysiological results showed that after priming with emotional faces (angry and happy), self-face elicited similar P3 amplitudes to friend-face in individuals with low self-esteem, but not in individuals with high self-esteem. The results suggest that as low self-esteem raises fears of social rejection and exclusion, priming with emotional faces (angry and happy) can weaken the SPA in low self-esteem individuals but not in high self-esteem individuals.

## Introduction

Self-face recognition denotes the process by which a person can recognize their own face by distinguishing it from another’s face. Individuals generally recognize their own face faster than another’s face, which is known as the self-face processing advantage (SPA) (Ma and Han, 2009, 2010). Recently, researchers have explored the electrophysiological response to the SPA more fully. Event-related potentials (ERPs), which originate as post-synaptic potentials, reflect the electrical activity in the brain in response to a specific event or stimulus (Luck, 2005). This technology has the advantage of a high temporal resolution, thus it has been widely used in cognitive psychology to investigate the time course of social cognitive processing, including the SPA (Sui et al., 2009; Tacikowski and Nowicka, 2010; Guan et al., 2014, 2015). Research has found that the electrophysiological indexes of the SPA – for example, the late P3 component (300–600 ms) over the posterior sites – are enhanced when responding to a self-face in comparison to other-faces (Gunji et al., 2009; Sui et al., 2009; Miyakoshi et al., 2010; Tacikowski and Nowicka, 2010; Guan et al., 2015).

Some interesting findings have emerged from recent studies that have investigated whether a social evaluation threat could influence the SPA. For example, the faces of faculty advisors, which would be regarded as a social threat for their graduate students, weakened the SPA by showing a faster response to their advisors’ faces than to their own faces (Ma and Han, 2009). Moreover, as a sign of negative evaluation and social threat, angry face eliminated the neural processing of SPA, showing that self-face elicited similar P3 amplitudes to friend-face (Guan et al., 2015).

It has long been suggested that a person’s self-esteem can be regarded as a threat buffer (Pyszczynski et al., 2004). For example, sociometer theory regards a person’s self-esteem system as a sociometer which reflects the degree of a person’s inclusion or exclusion by other people (Leary et al., 1995). As an interpersonal monitor, self-esteem could protect the person against social rejection and exclusion (Leary et al., 1995). Previous empirical studies have provided evidence that supports this theory. One study has reported that individuals with high self-esteem suffer less emotional distress when they encounter negative social evaluation than do individuals with low self-esteem (Brown, 2010). Moreover, high self-esteem individuals respond to evaluation threats by derogating others and emphasizing positive features of themselves, whereas individuals with low self-esteem respond to evaluation threats with self-degradation and increased social exclusion fears (Baldwin and Sinclair, 1996; Dodgson and Wood, 1998; Vohs and Heatherton, 2001). In addition, low self-esteem individuals show an enhanced attentional bias for social rejection facial expressions compared to high self-esteem individuals (Li et al., 2012). In conclusion, it seems that low self-esteem individuals are particularly sensitive to social rejection information whilst high self-esteem individuals can buffer the negative emotional consequences caused by social evaluation threats. However, it is rather surprising that little research has directly investigated the neural evidence of how self-esteem modulates the social evaluation threat to the SPA, which is of great significance for maintaining the mental health of low self-esteem individuals and helping them adapt to social life.

Emotional facial expressions are important social stimuli as well as effective social cues in interpersonal communication (Öhman, 1986). In the current study therefore, we used emotional facial expressions as the social evaluation information. After presenting prime faces (angry, happy, and neutral), both high and low self-esteem participants were asked to judge whether the target face (self, friend, and stranger) was familiar or unfamiliar. Meanwhile, their electrophysiological data in response to the target faces was recorded. We were interested in whether self-esteem could buffer the social evaluation threat to the SPA both in behavioral data and in the ERP component.

Besides negative social evaluation information (e.g., angry face), positive social evaluation information (e.g., happy face) may also be perceived distortedly by some people. For instance, patients with generalized social phobias (GSPs), who are sensitive to social interactions and disapproval by others (Stein et al., 2002), do not rate happy faces as approachable as healthy people do (Campbell et al., 2009). Participants with a high level of social anxiety tend to interpret positive social information as having a negative meaning (D’Argembeau et al., 2003), whilst low self-esteem individuals feel social rejection even after being told that they are personally accepted (Nezlek et al., 1997). As low self-esteem raises fears of social rejection and exclusion, these individuals may regard both positive evaluation information and negative evaluation information as a social evaluation threat. In the current study therefore, we hypothesized that both angry faces and happy faces would be more likely to weaken the SPA in low self-esteem participants compared to high self-esteem participants. We hypothesized further that, after priming with emotional faces (angry and happy), the SPA would be eliminated in low self-esteem individuals and thus self-face would elicit a similar P3 amplitude to other-face, whereas the SPA would persist for individuals with high self-esteem and thus self-face would elicit a larger P3 amplitude compared to other-face.

## Materials and Methods

### Ethics Statement

This study was approved by the Southwest University ethics review board. Prior to obtaining the written informed consents, a complete explanation of the study was given to all participants. All subjects gave written informed consent in accordance with the Declaration of Helsinki.

### Participants

Fifteen pairs of healthy Chinese undergraduates who were gender-matched friends and had known each other for more than 1 year were recruited in the study. Each pair of participants were from the same class, lived in the same dormitory and orally reported that they were good friends to each other. Three participants did not complete the formal experiment after being photographed, thus 27 participants (11 males, 16 females, mean age = 21.4 years, *SD* = 1.6 years) took part in this study as paid volunteers. In order to exclude the influence of depression, we enrolled those participants whose Beck depression inventory (BDI) scores were lower than 14. Participants were then divided into two groups: a high self-esteem group (SE score ≥ 32, 13 participants, five males, eight females, mean age = 20.9 years, *SD* = 1.4 years) and a low self-esteem group (SE score ≤ 31, 14 participants, six males, eight females, mean age = 21.8 years, *SD* = 1.8 years). Participants were divided according to their mean score as measured by the Rosenberg self-esteem (SE) scale. A paired *t*-test showed that the scores on the Rosenberg self-esteem scale were significantly different between the high self-esteem group and the low self-esteem group, *t*(1,25) = 7.914, *p* < 0.001. However, age (*p* = 0.184) was not significantly different between the two groups.

None of the participants had any previous experience with similar tasks. All participants were right-handed and had normal or corrected to normal visual acuity. All participants reported no history of, or currently suffered from, neurological or psychiatric disorders, significant physical illness, head injury, alcohol or drug abuse, or family history of psychiatric disorder or alcohol/drug abuse (as revealed by the participants’ self-reporting).

### Materials

As in our previous study (Guan et al., 2015), we selected eight angry, eight happy, and eight neutral faces from the Chinese Affective Face Picture System (CAFPS) as the prime faces (Lu et al., 2005). CAFPS was established in a similar way to the International Affective Picture System except that the models were all Chinese. CAFPS has been widely used in previous research (Duan et al., 2010; Luo et al., 2010; Zhang et al., 2015; Wang et al., 2016). There are four males and four females faces in each category. The mean valence of angry faces, happy faces and neutral faces on a 1–9 point scale was 2.64 ± 0.48, 6.96 ± 0.51, and 4.77 ± 0.34 respectively, and there are significant differences between each other, *F*(2,7) = 183.317, *p* < 0.001. Each prime face image was presented at 260 × 300 pixels.

Three types of target stimuli were used: self-face, friend-face, and stranger-face. Before the study, photographs were taken to include facial images of the participants, and images of the participants’ gender-matched friends and strangers (four females and four males). Each target face was presented at 260 × 300 pixels. To distinguish target faces from prime faces, all target faces displaying neutral facial expressions were oriented to both left and right sides, with five images at different angles of rotation for each side (15, 30, 45, 60, and 75°).

There were in total 960 trials presented randomly. After each prime face condition, 160 trials of familiar faces (self-face and friend-face, with 80 trials for each) and an equal number of trials of unfamiliar faces (stranger-face) were presented. Participants were asked to judge whether the target face was familiar (self and friend) or unfamiliar (stranger) in each trial (Tacikowski and Nowicka, 2010). There were eight rest periods throughout the whole process.

**FIGURE 1:**
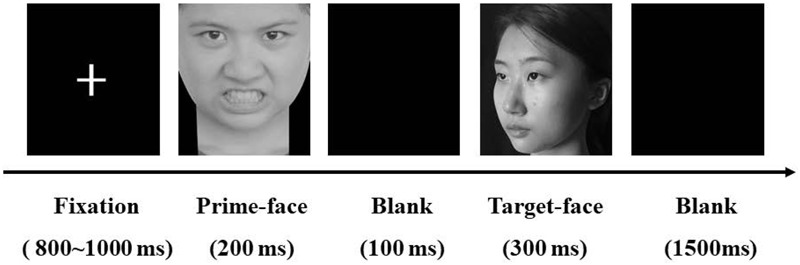
Illustration of the experimental stimuli and procedure.

Before each trial, a fixation sign was displayed randomly at the center of the screen with a duration of 800–1000 ms. A prime face then appeared for 200 ms followed by a blank screen with a duration of 100 ms. A target face was then displayed for 300 ms followed by a 1,500 ms blank screen for the participant to judge the familiarity of the target face by pressing keys with their index or middle finger (see **Figure 1**). To rule out lateral bias in the motor responses, participants responded with their left hand and right hand in different blocks, and the order effect was counterbalanced across participants.

### Procedure

Participants were asked to have lunch and to get adequate sleep before coming to lab. They were exposed to the test between 14:30 and 17:00. All participants refrained from physical exercise, smoking, eating, and drinking alcoholic beverages, coffee or tea at least 1 h prior to testing. Participants were given detailed instructions concerning the task upon arrival at the laboratory. They were asked to maintain focus on the fixation point during EEG recording. After completing the face-familiarity judgment task, participants were given a questionnaire using the Rosenberg self-esteem scale (RSE) to examine their self-esteem. RSE is a four-point scale (1: strongly disagree, 4: strongly agree) to assesses one’s overall evaluation of self-worth (Rosenberg, 1965). It is made up of 10 items, with the negative items needing to be reverse scored. Cronbach’s α for the RSE is 0.813 in this sample. Finally, participants were debriefed in detail about the purpose of the present experiment.

### EEG Recordings

The electroencephalogram (EEG) was recorded from 64 scalp sites using tin passive electrodes mounted on an elastic cap according to the 10–20 system positions with the reference on the left and right mastoids, using apparatus from Brain Products, Germany. The vertical and horizontal electrooculogram (EOG) was recorded from electrodes placed above and below the left eye, and at the right and left outer canthi, respectively. The inter-electrode impedance was maintained below 5 kΩ throughout the whole process. The EEG and EOGs were amplified by a bandpass of 0.05–100 Hz, and continuous sampling was conducted at 500 Hz/channel during online recording.

### Data Analysis

#### Behavioral Data Analysis

Three-way ANOVAs were conducted to analyze the response accuracies and reaction times separately with group (low self-esteem vs. high self-esteem) as a between-subject variable, and with prime face (angry vs. happy vs. neutral) and target face (self vs. friend vs. stranger) as the within-subject variables. All significant *p*-values were calculated using the Greenhouse–Geisser epsilon, and Bonferroni adjustment was used for multiple comparisons in *post hoc* tests.

#### ERP Data Analysis

Brain Vision Analyzer 1.05 (Brain Products, Germany) was used as the software for data analysis. The Gratton and Coles algorithm was used off-line for rejecting eye movement artifacts such as blinks and other movements (Gratton et al., 1983). Trials with artifacts contaminated by amplifier clippings, bursts of electromyographic (EMG) activity, or peak-to-peak deflections exceeding ± 80 μV at any electrode were excluded from the average.

As the participants’ mean reaction time for the target face was about 600 ms, we segmented ERP data into epochs from 500 ms before the target face onset to 600 ms after the target face onset. In order to accurately assess the influence of the prime face on the subsequent target face processing, we calculated the baseline activity between -500 and -300 ms, starting from 200 ms before the prime face onset (Guan et al., 2015). Previous studies have revealed that the P3 component over the time period of 300–600 ms at the posterior midline region reflects the time course of the SPA (Sui et al., 2009; Guan et al., 2014, 2015). In addition, voltage topographies in the present study showed larger P3 amplitudes over the posterior region; thus we analyzed the mean amplitude of the P3 component (300–600 ms) over the posterior region (CPz, Pz, POz electrodes).

The mean amplitudes of the P3 component were tested with a four-way ANOVA with group (low self-esteem vs. high self-esteem) as a between-subject variable, and with prime face (angry vs. happy vs. neutral), target face (self vs. friend vs. stranger) and electrodes (CPz, Pz, POz) as the within-subject variables. All significant *p*-values were calculated using the Greenhouse–Geisser epsilon, and Bonferroni adjustment was used for multiple comparisons in *post hoc* tests.

## Results

### Behavioral Data

**FIGURE 2:**
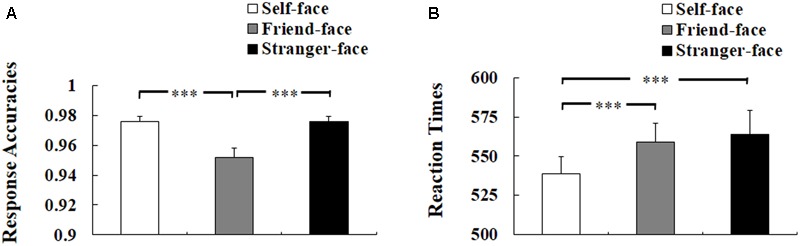
Behavioral results. **(A)** Response accuracies. **(B)** Reaction times (RTs). Error bars represent standard errors. ^∗^*p* < 0.05; ^∗∗^*p* < 0.01; ^∗∗∗^*p* < 0.001.

Three-way ANOVA of response accuracies showed a significant main effect of target face, *F*(2,50) = 29.708, *p* < 0.001, η^2^ = 0.543, suggesting worse response accuracies to friend-face compared with self-face and stranger-face (*ps* < 0.001), but the difference between self-face and stranger-face was not significant (*p* = 1) see **Figure 2A**). There was neither a main effect of self-esteem nor any interaction effect of self-esteem.

Three-way ANOVA of RTs for correct responses showed a significant main effect of target face, *F*(2,50) = 15.456, *p* < 0.001, η^2^ = 0.382, suggesting faster response to self-face compared with friend-face and stranger-face (*ps* < 0.001), but there was no significant difference between friend-face and stranger-face (*p* = 1) (see **Figure 2B**). There was neither a main effect of self-esteem nor any interaction effect of self-esteem.

### ERP Data

**FIGURE 3:**
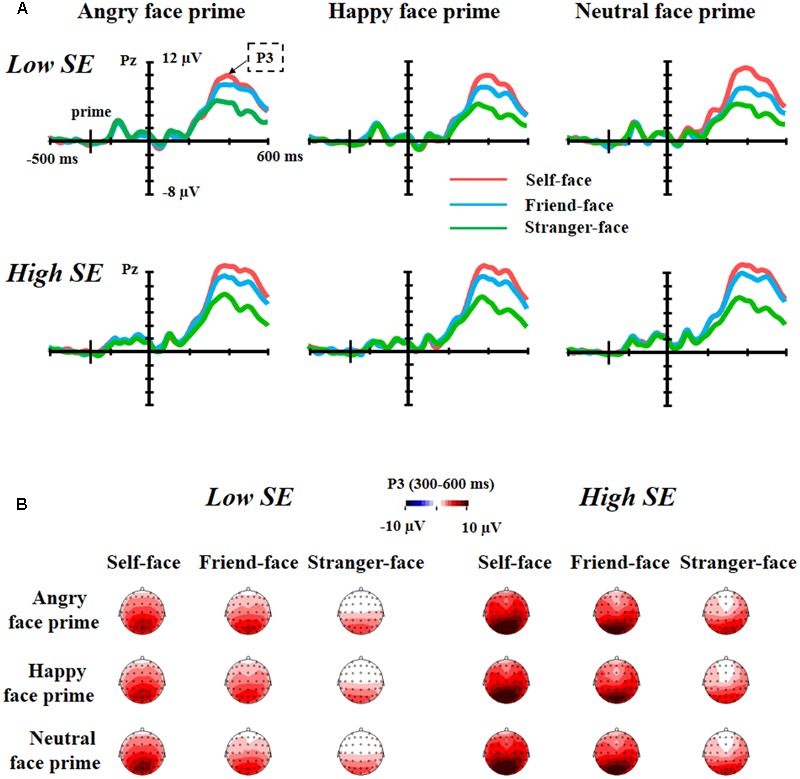
The grand average waveforms and top views of voltage topographies. **(A)** Grand average waveforms of self-face, friend-face and stranger-face after angry face prime, happy face prime and neutral face prime over Pz electrode position for both low self-esteem participants and high self-esteem participants. **(B)** Top views of voltage topographies of self-face, friend-face and stranger-face after angry face prime, happy face prime and neutral face prime for both low self-esteem participants and high self-esteem participants.

**Figure 3** showed the grand average waveforms and top views of voltage topographies of self-face, friend-face and stranger-face after angry face prime, happy face prime and neutral face prime for both low and high self-esteem participants.

#### Main Effects

Four-way ANOVA of the P3 amplitudes showed a significant main effect of electrodes, *F*(2,50) = 7.548, *p* < 0.01, η^2^ = 0.232, suggesting that larger amplitudes were elicited at Pz than POz and CPz (*ps* < 0.05), but there was no significant differences between CPz and POz (*p* = 0.574). Moreover, there was a significant main effect of group, *F*(2,50) = 4.286, *p* < 0.05, η^2^ = 0.146, suggesting that P3 amplitudes of high self-esteem participants were larger than that of low self-esteem participants. Furthermore, there was a significant main effect of prime face, *F*(2,50) = 4.296, *p* < 0.05, η^2^ = 0.147, suggesting that the target face elicited larger P3 amplitudes following angry prime face than that following happy prime face (*p* = 0.014), but there was neither significant differences between angry prime face and neutral prime face (*p* = 0.799), nor significant differences between happy prime face and neutral prime face (*p* = 0.120). At last, the main effect of target face was also significant, *F*(2,50) = 93.021, *p* < 0.001, η^2^ = 0.788, suggesting that self-face elicited larger P3 amplitudes compared with both friend-face and stranger-face (*ps* < 0.001), and friend-face elicited larger P3 amplitudes compared with stranger-face (*p* < 0.001).

#### Interaction Effects

**FIGURE 4:**
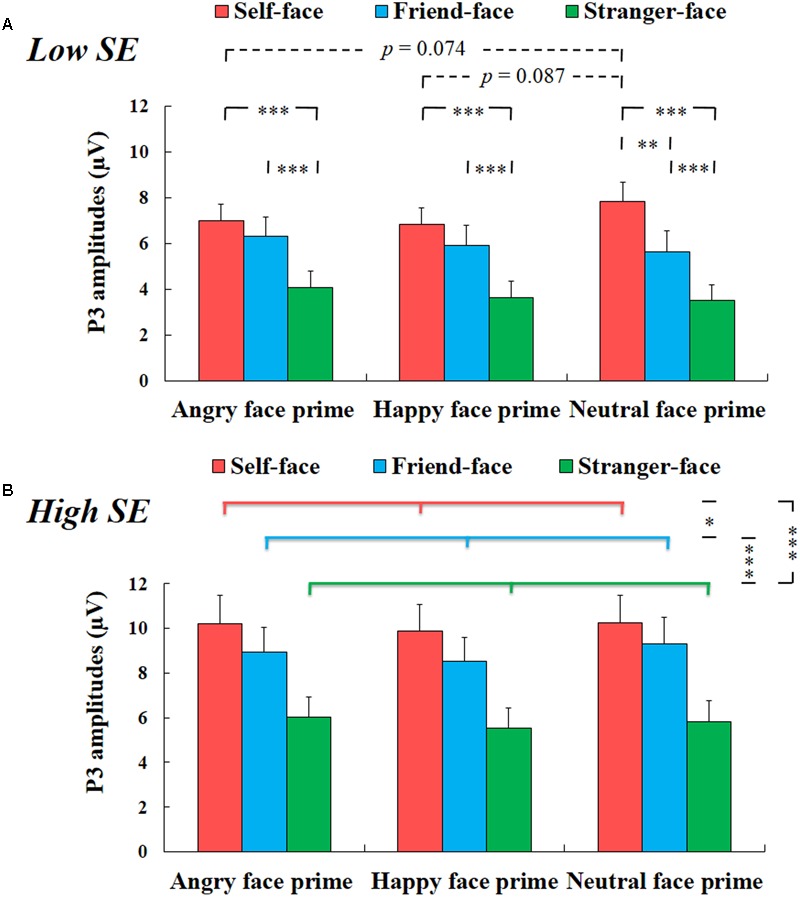
The mean amplitude results of P3 components. **(A)** Low self-esteem. Interaction effect between prime face and target face for low self-esteem participants (self-face, friend-face and stranger-face after angry face prime, happy face prime and neutral face prime over CPz, Pz, and POz electrode positions). **(B)** High self-esteem. Main effect of target face for high self-esteem participants (self-face, friend-face and stranger-face over CPz, Pz, and POz electrode positions). Error bars represent standard errors. ^∗^*p* < 0.05; ^∗∗^*p* < 0.01; ^∗∗∗^*p* < 0.001.

There was a significant interaction effect among group, prime face and target face, *F*(4,100) = 3.200, *p* < 0.05, η^2^ = 0.113. For high self-esteem participants, two-way repeated-measures ANOVA with prime face and target face only showed a significant main effect of target face, *F*(2,24) = 42.836, *p* < 0.001, η^2^ = 0.781, suggesting that the amplitudes of P3 for self-face were larger than that for friend-face and for stranger-face (*ps* < 0.05), and the amplitudes of P3 for friend-face were larger than that for stranger-face (*p* < 0.001) (see **Figure 4**). We did not find any other main effects or interaction effects.

For low self-esteem participants, two-way repeated-measures ANOVA also showed a significant main effect of target face, *F*(2,26) = 52.799, *p* < 0.001, η^2^ = 0.802, suggesting that the amplitudes of P3 were larger in response to self-face compared to friend-face and stranger-face (*ps* < 0.01), and the amplitudes of P3 were larger in response to friend-face than to stranger-face (*p* < 0.001). In addition, there was also a significant interaction effect between prime face and target face, *F*(4,52) = 6.066, *p* < 0.01, η^2^ = 0.318. After angry face prime, the P3 amplitude was larger in response to both self-face and friend-face compared to stranger face (*ps* < 0.001), while the P3 amplitude did not show significant differences between self-face identification and friend-face identification (*p* = 0.113). Following happy face prime, both self-face and friend-face elicited larger P3 amplitudes than stranger-face (*ps* < 0.001), while the P3 amplitude did not show significant differences between self-face and friend-face (*p* = 0.109). Following the neutral face prime, both self-face and friend-face elicited larger P3 amplitudes than stranger-face (*ps* < 0.001), and self-face elicited larger P3 amplitudes than friend-face (*p* < 0.01).

Our analyses also revealed that the P3 amplitude in response to self-face following the neutral face prime was marginal significant higher than that following the angry face prime (*p* = 0.074) and that following the happy face prime (*p* = 0.087), while it did not show significant difference between the angry face prime condition and the happy face prime condition (*p* = 1). However, the P3 amplitude neither showed a significant main effect of prime face in response to friend-face, *F*(2,26) = 3.002, *p* > 0.05, η^2^ = 0.188, nor showed a significant effect of prime face in response to stranger-face, *F*(2,26) = 3.358, *p* > 0.05, η^2^ = 0.205 (see **Figure 4**).

## Discussion

The aim of this study was to investigate the neural evidence of how self-esteem modulates the social evaluation threat to the SPA. Electrophysiological results indicate that priming with emotional faces weakened the P3 component in response to self-face processing in low self-esteem participants, whereas the SPA persisted for participants with high self-esteem.

Sociometer theory regards self-esteem as an interpersonal monitor, the function of which is to protect the person against social rejection and exclusion (Leary et al., 1995). Many studies have shown that low self-esteem individuals are often extremely sensitive to social rejection information (Sommer and Baumeister, 2002; Brown, 2010; Richter and Ridout, 2011). They devote attentional resources to potential rejection information (Dandeneau and Baldwin, 2004, 2009) and release more cortisol when reacting to rejection (Ford and Collins, 2010). ERP studies suggest that low self-esteem individuals have an enhanced attentional bias for facial expressions of social rejection compared with high self-esteem individuals (Li et al., 2012). The results of the current electrophysiological study were consistent with previous findings, which show that angry face, as being representative of negatively evaluated faces and of social rejection, weakens the SPA (self vs. friend) in low self-esteem participants.

Interestingly, although happy faces are usually regarded as expressing approval, our results show that priming with happy faces can also weaken the SPA (self vs. friend) in low self-esteem participants. In addition to negative social evaluation information (e.g., angry face), positive social evaluation information (e.g., happy face) may also be perceived distortedly by some people. For example, an fMRI study indicated that patients with social phobias show comparable neural activity in response to happy and angry facial expressions (Straube et al., 2005). Participants with a high level of social anxiety also showed increased avoidance tendencies for both angry and happy facial expressions. Researchers have argued that, because patients with social anxiety disorder (SAD) fear social interaction, the fear of being invited into further contact may account for the avoidance of happy face (Heuer et al., 2007; Roelofs et al., 2010). Although low self-esteem individuals and social anxiety individuals have different sorts of mental problems, they are all sensitive to social interactions that may result in exclusion by others. According to sociometer theory, low self-esteem individuals are sensitive to interpersonal interaction caused by the fear of social rejection and exclusion (Leary et al., 1995). Both happy face and angry face are important cues for social interaction. Low self-esteem individuals are therefore also excessively sensitive to happy face, which might lead to the result that happy face weakens the self-face advantage in low self-esteem individuals.

Our previous study found that priming with angry face elicited larger P3 amplitudes of target face compared to those after priming with happy face (Guan et al., 2015). The current results are consistent with these studies, which indicate that P3 separates prime emotional valence and shows a negativity prime bias (angry > happy). Moreover, the P3/late positive components (LPC) has also been considered to be an index of self-processing specificity (Tacikowski and Nowicka, 2010) and shows enhanced amplitude in recognizing, firstly, self-face (Gunji et al., 2009; Sui et al., 2009; Miyakoshi et al., 2010; Tacikowski and Nowicka, 2010; Guan et al., 2014, 2015); secondly, self-name (Berlad and Pratt, 1995; Folmer and Yingling, 1997; Perrin et al., 1999; Tacikowski and Nowicka, 2010; Zhao et al., 2011); thirdly, self-object (Miyakoshi et al., 2007); fourthly, self-handwriting (Chen et al., 2008); and fifthly, autobiographical information (Gray et al., 2004). Similarly, our results indicate that P3 showed enhanced response to self-face compared to both friend-face and stranger-face, which reflected the specificity of self-processing.

Previous research had proposed that P3 amplitude was negatively associated with the cognitive load of task (Polich and Kok, 1995; Kok, 2001). The current results showed an enhanced P3 response to both self-face and friend-face compared to stranger-face, which may reflect the lower cognitive load in processing familiar faces (both self-face and friend-face) compared to stranger-face. Moreover, cognitive load depends in part on the cognitive capacity of individuals (Polich and Kok, 1995; Kok, 2001; Polich, 2007). It seems that larger P3 amplitudes in high self-esteem individuals relative to low self-esteem individuals may imply their differences in cognitive capacity. Because low self-esteem individuals are not good at dealing with the situation of social interaction (Leary et al., 1995), the cognitive load of assessing the relation between social evaluation information and target-face was greater in low self-esteem individuals whose P3 amplitudes were reduced.

In addition, our results are consistent with previous research in finding that the advantage of self-face compared to familiar-face was eliminated (Ma and Han, 2009, 2010; Guan et al., 2014, 2015), whilst the advantage of self-face compared to stranger-face was difficult to eliminate (Guan et al., 2014, 2015). Familiar relationships could pose a greater threat to self-evaluation compared with non-familiar relationships when others outperform oneself on a highly self-related task (Tesser et al., 1988), which suggests that non-familiar relationships are less likely to affect the self-processing advantage. Therefore, the relative advantage of self-processing might be eliminated more easily when compared to a friend rather than to a stranger.

Some limitations of the present study should be addressed in future work. Firstly, we found that the emotional face prime exhibited its effect only by measuring a person’s electrophysiological response; measuring their behavioral response did not produce such a finding, which might reflect the sensitivity of electrophysiological recording. Secondly, although both happy faces and angry faces weakened the self-face processing in low self-esteem, it is not known from the present study whether the mechanism that links low self-esteem with the processing of happy faces is similar to the mechanism that links it with the processing of angry faces. Future studies should address this question in more detail. Thirdly, given the small sample size of the current study, we could not test the influence of other factors, such as gender difference.

## Conclusion

Our results indicate that as low self-esteem individuals are sensitive to both negative and positive social evaluation information caused by the fear of social rejection and exclusion, priming with emotional faces could weaken the SPA in low self-esteem individuals but not in high self-esteem individuals. Overall, our findings provide evidence that self-esteem modulates the effect of processing social evaluation information on the self-processing, which has potential clinical applications.

## Author Contributions

LG conceived this study and was involved in conducting the experiments, processing data, and writing the manuscript. YZ participated in data interpretation and writing the manuscript. YW participated in writing the manuscript. YC participated in processing data. JY was involved in conceiving this study, data interpretation and writing the manuscript.

## Conflict of Interest Statement

The authors declare that the research was conducted in the absence of any commercial or financial relationships that could be construed as a potential conflict of interest.

## References

[B1] Baldwin M. W. Sinclair L. (1996). Self-esteem and “if… then” contingencies of interpersonal acceptance. *J. Pers. Soc. Psychol.* 71 1130–1141. 10.1037/0022-3514.71.6.1130 8979382

[B2] Berlad I. Pratt H. (1995). P300 in response to the subject’s own name. *Electroencephalogr. Clin. Neurophysiol.* 96 472–474. 10.1016/0168-5597(95)00116-A 7555920

[B3] Brown J. D. (2010). High self-esteem buffers negative feedback: once more with feeling. *Cogn. Emot.* 24 1389–1404. 10.1080/02699930903504405

[B4] Campbell D. Sareen J. Stein M. Kravetsky L. Paulus M. Hassard S. (2009). Happy but not so approachable: the social judgments of individuals with generalized social phobia. *Depress. Anxiety* 26 419–424. 10.1002/da.20474 19242987

[B5] Chen A. Weng X. Yuan J. Lei X. Qiu J. Yao D. (2008). The temporal features of self-referential processing evoked by Chinese handwriting. *J. Cogn. Neurosci.* 20 816–827. 10.1162/jocn.2008.20505 18201135

[B6] Dandeneau S. D. Baldwin M. W. (2004). The inhibition of socially rejecting information among people with high versus low self-esteem: the role of attentional bias and the effects of bias reduction training. *J. Soc. Clin. Psychol.* 23 584–603. 10.1521/jscp.23.4.584.40306

[B7] Dandeneau S. D. Baldwin M. W. (2009). The buffering effects of rejection-inhibiting attentional training on social and performance threat among adult students. *Contemp. Educ. Psychol.* 34 42–50. 10.1016/j.cedpsych.2008.05.004

[B8] D’Argembeau A. Van der Linden M. Etienne A.-M. Comblain C. (2003). Identity and expression memory for happy and angry faces in social anxiety. *Acta Psychol.* 114 1–15. 10.1016/S0001-6918(03)00047-7 12927340

[B9] Dodgson P. G. Wood J. V. (1998). Self-esteem and the cognitive accessibility of strengths and weaknesses after failure. *J. Pers. Soc. Psychol.* 75 178–197. 10.1037/0022-3514.75.1.178 9686458

[B10] Duan X. Dai Q. Gong Q. Chen H. (2010). Neural mechanism of unconscious perception of surprised facial expression. *Neuroimage* 52 401–407. 10.1016/j.neuroimage.2010.04.021 20398771

[B11] Folmer R. L. Yingling C. D. (1997). Auditory P3 responses to name stimuli. *Brain Lang.* 56 306–311. 10.1006/brln.1997.1828 9027376

[B12] Ford M. B. Collins N. L. (2010). Self-esteem moderates neuroendocrine and psychological responses to interpersonal rejection. *J. Pers. Soc. Psychol.* 98 405–419. 10.1037/a0017345 20175621

[B13] Gratton G. Coles M. G. Donchin E. (1983). A new method for off-line removal of ocular artifact. *Electroencephalogr. Clin. Neurophysiol.* 55 468–484. 10.1016/0013-4694(83)90135-9 6187540

[B14] Gray H. M. Ambady N. Lowenthal W. T. Deldin P. (2004). P300 as an index of attention to self-relevant stimuli. *J. Exp. Soc. Psychol.* 40 216–224. 10.1016/0168-5597(95)00116-A

[B15] Guan L. Qi M. Li H. Hitchman G. Yang J. Liu Y. (2015). Priming with threatening faces modulates the self-face advantage by enhancing the other-face processing rather than suppressing the self-face processing. *Brain Res.* 1608 97–107. 10.1016/j.brainres.2015.03.002 25765156

[B16] Guan L. Qi M. Zhang Q. Yang J. (2014). The neural basis of self-face recognition after self-concept threat and comparison with important others. *Soc. Neurosci.* 9 424–435. 10.1080/17470919.2014.920417 24852316

[B17] Gunji A. Inagaki M. Inoue Y. Takeshima Y. Kaga M. (2009). Event-related potentials of self-face recognition in children with pervasive developmental disorders. *Brain Dev.* 31 139–147. 10.1016/j.braindev.2008.04.011 18590948

[B18] Heuer K. Rinck M. Becker E. S. (2007). Avoidance of emotional facial expressions in social anxiety: the approach–avoidance task. *Behav. Res. Ther.* 45 2990–3001. 10.1016/j.brat.2007.08.010 17889827

[B19] Kok A. (2001). On the utility of P3 amplitude as a measure of processing capacity. *Psychophysiology* 38 557–577. 10.1017/S0048577201990559 11352145

[B20] Leary M. R. Tambor E. S. Terdal S. K. Downs D. L. (1995). Self-esteem as an interpersonal monitor: the sociometer hypothesis. *J. Pers. Soc. Psychol.* 68 518–530. 10.1037/0022-3514.68.3.518

[B21] Li H. Zeigler-Hill V. Yang J. Jia L. Xiao X. Luo J. (2012). Low self-esteem and the neural basis of attentional bias for social rejection cues: evidence from the N2pc ERP component. *Pers. Indiv. Differ.* 53 947–951. 10.1016/j.paid.2012.03.004

[B22] Lu B. Hui M. Yu-Xia H. (2005). The development of native Chinese affective picture system–a pretest in 46 college students. *Chin. Ment. Health J.* 19 719–722.

[B23] Luck S. J. (2005). *An Introduction to the Event-Related Potentials Technique.* Cambridge: MIT Press.

[B24] Luo W. Feng W. He W. Wang N. Y. Luo Y. J. (2010). Three stages of facial expression processing: ERP study with rapid serial visual presentation. *Neuroimage* 49 1857–1867. 10.1016/j.neuroimage.2009.09.018 19770052 PMC3794431

[B25] Ma Y. Han S. (2009). Self-face advantage is modulated by social threat–Boss effect on self-face recognition. *J. Exp. Soc. Psychol.* 45 1048–1051. 10.1016/j.jesp.2009.05.008

[B26] Ma Y. Han S. (2010). Why we respond faster to the self than to others? An implicit positive association theory of self-advantage during implicit face recognition. *J. Exp. Psychol. Hum. Percept. Perform.* 36 619–633. 10.1037/a0015797 20515192

[B27] Miyakoshi M. Kanayama N. Iidaka T. Ohira H. (2010). EEG evidence of face-specific visual self-representation. *Neuroimage* 50 1666–1675. 10.1016/j.neuroimage.2010.01.030 20079852

[B28] Miyakoshi M. Nomura M. Ohira H. (2007). An ERP study on self-relevant object recognition. *Brain Cogn.* 63 182–189. 10.1016/j.bandc.2006.12.001 17223240

[B29] Nezlek J. B. Kowalski R. M. Leary M. R. Blevins T. Holgate S. (1997). Personality moderators of reactions to interpersonal rejection: depression and trait self-esteem. *Pers. Soc. Psychol. Bull.* 23 1235–1244. 10.1177/01461672972312001

[B30] Öhman A. (1986). Face the beast and fear the face: animal and social fears as prototypes for evolutionary analyses of emotion. *Psychophysiology* 23 123–145. 10.1111/j.1469-8986.1986.tb00608.x 3704069

[B31] Perrin F. Garcìa-Larrea L. Mauguière F. Bastuji H. (1999). A differential brain response to the subject’s own name persists during sleep. *Clin. Neurophysiol.* 110 2153–2164. 10.1016/S1388-2457(99)00177-7 10616121

[B32] Polich J. (2007). Updating P300: an integrative theory of P3a and P3b. *Clin. Neurophysiol.* 118 2128–2148. 10.1016/j.clinph.2007.04.019 17573239 PMC2715154

[B33] Polich J. Kok A. (1995). Cognitive and biological determinants of P300: an integrative review. *Biol. psychol.* 41 103–146. 10.1016/0301-0511(95)05130-9 8534788

[B34] Pyszczynski T. Greenberg J. Solomon S. Arndt J. Schimel J. (2004). Why do people need self-esteem? A theoretical and empirical review. *Psychol. Bull.* 130 435–468. 10.1037/0033-2909.130.3.435 15122930

[B35] Richter A. Ridout N. (2011). Self-esteem moderates affective reactions to briefly presented emotional faces. *J. Res. Pers.* 45 328–331. 10.1016/j.jrp.2011.02.008

[B36] Roelofs K. Putman P. Schouten S. Lange W.-G. Volman I. Rinck M. (2010). Gaze direction differentially affects avoidance tendencies to happy and angry faces in socially anxious individuals. *Behav. Res. Ther.* 48 290–294. 10.1016/j.brat.2009.11.008 19962692

[B37] Rosenberg M. (1965). *Society and the Adolescent Self-Image.* Princeton, NJ: Princeton University Press.

[B38] Sommer K. L. Baumeister R. F. (2002). Self-evaluation, persistence, and performance following implicit rejection: the role of trait self-esteem. *Pers. Soc. Psychol. Bull.* 28 926–938. 10.1177/014616720202800706

[B39] Stein M. B. Goldin P. R. Sareen J. Zorrilla L. T. E. Brown G. G. (2002). Increased amygdala activation to angry and contemptuous faces in generalized social phobia. *Arch. Gen. Psychiatry* 59 1027–1034. 10.1001/archpsyc.59.11.1027 12418936

[B40] Straube T. Mentzel H.-J. Miltner W. H. (2005). Common and distinct brain activation to threat and safety signals in social phobia. *Neuropsychobiology* 52 163–168. 10.1159/000087987 16137995

[B41] Sui J. Liu C. H. Han S. (2009). Cultural difference in neural mechanisms of self-recognition. *Soc. Neurosci.* 4 402–411. 10.1080/17470910802674825 19739032 PMC3348608

[B42] Tacikowski P. Nowicka A. (2010). Allocation of attention to self-name and self-face: an ERP study. *Biol. Psychol.* 84 318–324. 10.1016/j.biopsycho.2010.03.009 20298741

[B43] Tesser A. Millar M. Moore J. (1988). Some affective consequences of social comparison and reflection processes: the pain and pleasure of being close. *J. Pers. Soc. Psychol.* 54 49–61. 10.1037/0022-3514.54.1.49 3346807

[B44] Vohs K. D. Heatherton T. F. (2001). Self-Esteem and threats to self: implications for self-construals and interpersonal perceptions. *J. Pers. Soc. Psychol.* 81 1103–1118. 10.1037/0022-3514.81.6.1103 11761311

[B45] Wang X. Fang Y. Cui Z. Xu Y. He Y. Guo Q. (2016). Representing object categories by connections: evidence from a multivariate connectivity pattern classification approach. *Hum. Brain Mapp.* 37 3685–3697. 10.1002/hbm.22636 27218306 PMC6867288

[B46] Zhang W. Li H. Pan X. (2015). Positive and negative affective processing exhibit dissociable functional hubs during the viewing of affective pictures. *Hum. Brain Mapp.* 36 415–426. 10.1002/hbm.22636 25220389 PMC6869282

[B47] Zhao K. Wu Q. Zimmer H. D. Fu X. (2011). Electrophysiological correlates of visually processing subject’s own name. *Neurosci. Lett.* 491 143–147. 10.1016/j.neulet.2011.01.025 21256923

